# Reversal of NADPH Oxidase-Dependent Early Oxidative and Inflammatory Responses in Chronic Obstructive Pulmonary Disease by Puerarin

**DOI:** 10.1155/2022/5595781

**Published:** 2022-04-25

**Authors:** Pan Zhang, Yixuan Zhang, Lu Wang, Xinjing Wang, Shiqing Xu, Zhenguo Zhai, Chen Wang, Hua Cai

**Affiliations:** ^1^Department of Pulmonary and Critical Care Medicine, China-Japan Friendship Hospital, Beijing University of Chinese Medicine, Beijing, China; ^2^Division of Molecular Medicine, Department of Anesthesiology, Division of Cardiology, Department of Medicine, David Geffen School of Medicine, University of California Los Angeles, Los Angeles, CA, USA; ^3^Institute of Respiratory Medicine, Chinese Academy of Medical Sciences and Peking Union Medical College, Beijing, China

## Abstract

In the present study, we investigated effects of Puerarin on the early oxidative and inflammatory responses in the lung triggered by acute cigarette smoking (ACS). C57BL/6 mice were exposed to ACS for 1 hr in the presence or absence of Puerarin and harvested at 2, 6, and 24 hours. ACS induced significant increases in superoxide production in mouse lungs at 2 and 6 hours; and superoxide production was also elevated in a time and concentration dependent manner in cigarette smoke extract (CSE) stimulated human small airway epithelial cells (HSAECs), which was dose-dependently abrogated by Puerarin. ACS exposure upregulated NOX1, NOX2, and NOX4 protein expression in mouse lungs. Likewise, NOX1 and NOX4 were upregulated in CSE-stimulated HSAECs. These responses were significantly or completely attenuated by Puerarin. ACS induced significant infiltrations of neutrophils and macrophages in mouse lung parenchyma and BAL fluid, which were completely or significantly abrogated by Puerarin, so was the activation of the NF-*к*B pathway and the upregulation in inflammatory mediators including TNF-*α*, KC (murine homolog of IL-8), COX-2, IL-6 and MCP-1. Nuclear translocation of p65, IL-8 secretion, and upregulation of COX-2 in CSE stimulated HSAECs were also markedly attenuated by Puerarin. Moreover, ACS or CSE stimulated upregulation in reactive oxygen species (ROS) production and expression of inflammatory mediators were alleviated by ROS scavenger TEMPO in vivo and vitro, with no synergy combining with Puerarin, indicating that the effects of Puerarin are redox-sensitive following activation of NOX. In summary, our data for the first time demonstrate that Puerarin robustly attenuates NOX isoform-dependent ROS production and inflammatory activation in ACS exposed mice and CSE treated HSAECs, indicating that Puerarin might be used as a robust therapeutic agent for early or early stage COPD.

## 1. Introduction

Chronic obstructive pulmonary disease (COPD), represented in the form of chronic bronchitis and emphysema, is associated with progressive and irreversible airflow limitation. It is the third leading cause of death worldwide with high morbidity and mortality [1]. The 2015 study of Global Burden of Diseases, Injuries, and Risk Factors reported that the number of COPD cases increased by 44.2% to 174.5 million from 1990 to 2015 [2]. However, the underlying molecular mechanisms of COPD remain incompletely understood with limited therapeutic options. Recent work has implicated that “early stage COPD,” defined as disease stage at GOLD I and GOLD II, or “early COPD,” defined as “pre-disease” stage with decline in pulmonary function before GOLD I, represents a good therapeutic window, targeting of which is anticipated to be highly effective to prevent continuous progression of the disease [3]. Our current study focuses on the initiating phase of COPD induced by acute cigarette smoking (ACS) and the potential therapeutic value of Puerarin, an active ingredient of the Traditional Chinese Medicine (TCM) Gegen.

Cigarette smoking (CS) is a major causal factor for COPD, which is known to increase reactive oxygen species (ROS) production and inflammatory responses [4]. Even ACS exposure could result in rapid responses of oxidative stress and lung inflammation [5], while oxidative stress has been recognized as the key player in driving progression of COPD. Nonetheless, detailed molecular mechanisms as to what oxidase systems are activated to produce ROS to initiate early pathological processes of COPD, have remained unclear. Nicotinamide adenine dinucleotide phosphate (NAPDH) oxidase (NOX) family is the major enzymatic system involved in the basal and excessive ROS production implicated in cardiovascular physiology and pathogenesis of cardiovascular diseases [6–9]. Of note, redox-sensitive activation of NF-*κ*B regulates expression of important inflammatory mediators in COPD [10], such as tumor necrosis factor-alpha (TNF-*α*) [11], COX-2 [12], and IL-8 [13]. In this study, we aimed to examine roles of NOX isoforms and their downstream inflammatory effectors in mediating ACS induced inflammation, which drives development of early COPD. Moreover, cigarette smoking induced inflammation in small airways [14] (less than 2 mm in diameter) has been considered a primary and original site of injury to cause remodeling and obstruction of the airway in COPD [15], and small airway disease represents an important pathological feature in early stage COPD [16] where the rate of FEV1 decline is the greatest in GOLD I and GOLD II patients [17]. Hence, cigarette smoke extract (CSE) stimulated human small airway epithelial cells (HSAECs) can be used as a good model system in vitro for mechanistic studies of COPD. In the present study, ACS exposed mice and CSE stimulated HSAECs were examined to investigate the potential therapeutic effects of Puerarin on the early oxidative and inflammatory responses that are critical during the initiating phase of the COPD development.

Pueraria lobata (Willd.) Ohwi (Gegen) has been used to relieve fever and diarrhea in TCM practice. Puerarin (daidzein-8-C-glucoside-7, 4′-dihydroxy-8-C-glucosylisoflavone) is the key active component in Gegen, which has been shown to exert anti-inflammatory and anti-oxidative effects especially in cardiovascular diseases, cerebrovascular diseases, and diabetes as reviewed by Yang et al. [18]. Moreover, Puerarin was reported to alleviate ovalbumin (OVA) induced allergic inflammation in a mouse model of allergic asthma [19]. Wang and colleagues have also shown that Puerarin alleviates inflammation in LPS-induced acute lung injury [20]. However, the potential effects of Puerarin on ACS and CSE induced oxidative and inflammatory responses in vivo and in vitro, which can trigger initial development of early or early stage COPD, have remained unknown. In the present study, we examined early oxidative and inflammatory responses in ACS exposed mice and CSE stimulated HSAECs, and the potential reversal effects of Puerarin. We found that Puerarin markedly attenuated ACS induced upregulation in NOX1, 2, and 4 protein expression, ROS production, and lung inflammatory responses in mice. ACS exposure increased inflammatory cell counts in lung parenchyma and bronchoalveolar lavage fluid, activated NF-кB pathway, and upregulated expression of TNF-*α*, KC (murine homolog of IL-8), COX-2, IL-6, and MCP-1, all of which were alleviated by Puerarin. In CSE treated HSAECs, Puerarin dose-dependently decreased upregulation of NOX1 and NOX4, resulting in inhibition of ROS production, inactivation of NF-кB pathway, and abrogated IL-8 release and COX-2 upregulation. Our data for the first time demonstrate that Puerarin can potentially serve as a novel therapeutic agent for early or early stage COPD, via attenuation of ACS induced early oxidative and inflammatory responses in the mouse lung and small airway epithelial cells.

## 2. Material and Methods

### 2.1. Chemicals and Reagents

Puerarin was purchased from Sun Chemical Technology Co., Ltd. (E100180, Shanghai, China). TEMPO, dihydroethidium (DHE), dichloro-dihydro-fluorescein diacetate (DCFH-DA), and other chemicals used for preparation of Krebs-Henseleit bicarbonate buffer (KHB), Krebs-Ringer buffer (KRGB), and protein lysis buffer were purchased from MilliporeSigma (Louis, MO, USA) in the highest purity. Antibodies and dilutions used in immunohistochemistry, immunocytochemistry, and Western blotting analyses were summarized in supplemental Table 1.

### 2.2. Animal Studies

Male C57BL/6 mice (6-8 weeks old) were purchased from Charles River Laboratories (Beijing Branch, Beijing, China) and housed in specific pathogen-free (SPF) conditions with free access to food and water. The study was approved by the Animal Care Review Committee of the China-Japan Friendship Hospital (Beijing, China). The animals were randomly divided into 6 groups: (1) mice exposed to air as normal control; (2) mice exposed to acute cigarette smoking (ACS) using the standard TE-10B smoking machine (Teague Enterprise, Woodland, CA, USA) and further divided into time-dependent groups of 2, 6, or 24 hours post-ACS exposure; (3, 4) mice treated with ACS and two different doses of Puerarin (40, 80 mg/kg, respectively); (5) mice treated with ACS and TEMPO (100 mg/kg); and (6) mice treated with ACS and Puerarin plus TEMPO. Puerarin and TEMPO were administrated by intraperitoneal injection (i.p.) 1 hour before and after ACS exposure. The doses of Puerarin [20] and TEMPO [21] were selected based on existing literatures.

### 2.3. Acute Cigarette Smoking Exposure

Mice were exposed to acute cigarette smoking (ACS) using a whole body exposure chamber as part of the standard TE-10B smoking machine [22] (Model TE-10B, Teague Enterprises, Woodland, CA, USA) and commercial cigarettes (11 mg of tar and 1.1 mg of nicotine per cigarette, Trade name: Hongtashan, Hongta Tobacco Company Limited, China, one of the most popular brands of cigarettes with highest sales in China). The cigarette smoking machine was adjusted to produce a mixture of mainstream and sidestream smoke by burning eight cigarettes at one time. Each smoldering cigarette was puffed for 2 seconds, once every minute for a total of 8 puffs, at a flow rate of 1.05 l/min, to provide a standard puff of 35 cm^3^. The chamber atmosphere was monitored for total suspended particulates that stabilized at 150 mg/m^3^. Upon harvest, the left lung was perfused and formalin fixed for histological evaluation. The upper lobe of right lung was perfused and embedded in optimal cutting temperature (OCT) compound for fluorescent imaging analyses; the other 3 lobes of right lung were stored at -80°C for consequent analyses of mRNA and protein expression.

### 2.4. Measurement of ROS Production

Superoxide production in lung tissues was detected using dihydroethidium (DHE) fluorescent imaging. Freshly isolated lung tissues were embedded in optimal cutting temperature (OCT) compound, frozen at -20°C, and sectioned immediately. The sections (5 *μ*m) were then incubated with DHE (2 *μ*M; D7008, MilliporeSigma, St. Louis, MO, USA) for 30 min in the dark and washed with KHB buffer (118.0 mM NACl, 24.0 mM NaHCO3, 4.7 mM KCl, 1.2 mM KH2PO4, 1.2 mM MgSO4, 1.7 mM CaCl2, 10.0 mMglucose, 118.0 mMNACl, 24.0 mM NaHCO3) for 3 times. After washing, the sections were mounted, and the fluorescent images were captured using a Nikon A1R Confocal Microscope (Tokyo, Japan) at the wavelengths of 488/543 nm for excitation and emission, respectively. To examine whether Puerarin directly scavenges superoxide radcial via chemical reaction, different doses of Puerarin (20, 40, 80 *µ*M) were mixed with superoxide generator Paraquat (100 *µ*M, MilliporeSigma, Louis, MO, USA) in a cell free buffer system, and changes in superoxide production were determined using electron spin resonance (ESR) spectrophotometer (eScan, Bruker) as we previously published [6–9].

To examine superoxide production in CSE stimulated HSAECs, cells grown to confluence on coverslips in 24 well plates were washed three times with KHB buffer and incubated with DHE (2 *μ*M) for 30 min in the dark. The cells were then washed with ice cold KHB buffer for 3 times. After washing, the cells were mounted, and the fluorescent images were captured using a Nikon A1R Confocal Microscope (Tokyo, Japan) with the wavelengths of 488/543 nm for excitation and emission, respectively.

In parallel experiments, intracellular hydrogen peroxide levels in CSE exposed HSAECs were determined using 2,7-dichlorofluoroscein diacetate fluorescent probe (DCFH-DA; D6883, MilliporeSigma, St. Louis, MO, USA). Briefly, HSAECs grown to confluence on coverslips were incubated with DCFH-DA solution (20 *μ*M) for 30 min in the dark, and then washed with ice cold KRGB buffer (145 mM NaCl, 5.7 mM Na2HPO4, 4.86 mM KCl, 5.5 mM D-Glucose, 0.54 mM CaCl2, 1.22 mM MgSO4) for 3 times. After washing, the cells were mounted, and the fluorescent images were captured using a Nikon A1R Confocal Microscope (Tokyo, Japan), with excitation and emission wavelengths of 485/530 nm, respectively. All of the images were quantified using the NIH ImageJ software.

### 2.5. Hematoxylin and Eosin (H&E) Staining

Freshly isolated mouse left lungs (which had not been lavaged) were perfused and fixed with 10% neutral buffered formalin (MilliporeSigma, Louis, MO, USA), then paraffin embedded and cut into 5 *μ*m sections. Deparaffinized and rehydrated tissue sections were stained with Harris's hematoxylin for 9 min and then differentiated in 0.1% acid alcohol for 30 sec followed by counterstaining with eosin for 7 min and dehydration. The images were captured using a BX51 fluorescent microscope (Olympus, Tokyo, Japan).

### 2.6. Bronchoalveolar Lavage Cell Count

Airway inflammation was assessed by differential count of inflammatory cells in bronchoalveolar lavage (BAL) fluid. For collection of BALF at 2, 6, and 24 hours after ACS exposure, mice were anaesthetized by intraperitoneal injection of sodium pentobarbital (60 mg/kg). After partial excision of the trachea, a plastic cannula was inserted, and the airway was washed twice with 0.5 mL phosphate-buffered saline (PBS) at 37°C. The rib cage was then gently massaged to maximize the number of cells carefully collected by aspiration. This procedure was performed 6 times. The saline lavage was centrifuged at 200 × g at 4°C for 10 min. The supernatants were decanted, and the cell pellets were resuspended in 300 *μ*l of saline, kept at 4°C until cell counting using PlasMET counting chamber slides. The differential inflammatory cell counts in BALF were calculated using standard morphological criteria in the representative slide (minimum of 300 cells per slide) which were stained with Giemsa (Solarbio, Beijing, China) according to manufacturer's instructions.

### 2.7. RT-PCR Determination of mRNA Expression of Inflammatory Mediators

Total RNA of the lung tissues was isolated using TRIzol Reagent (Invitrogen, Carlsbad, CA, USA) and reversed to cDNA using High-Capacity cDNA Reverse Transcription Kit (Invitrogen, Carlsbad, CA, USA). Subsequently, PCR was performed using SsoFast Evagreen supermix (Bio-Rad Laboratories, Hercules, CA, USA) on a CFX96 Real-Time System (Bio-Rad Laboratories, Hercules, CA, USA) according to the manufacturer's instructions. The cycle step of reverse transcription (RT) and polymerase chain reaction (PCR) were performed following manufacturer's instructions. The results were normalized to *β*-actin. Primers used for each gene are presented in the supplemental Table 2.

### 2.8. Cell Culture and Treatments

Three independent batches of human small airway epithelial cells (HSAECs) were purchased from Lonza Bioscience (CC-2547, Walkersville, MD, USA), which were isolated from three healthy donors, and the information of the donors is presented in the supplemental Table 3. The cells were cultured in the small airway growth medium (SAGM) supplemented with growth factors (Lonza Bioscience, Walkersville, MD, USA) following manufacturer's instructions. HSAECs between passage three and seven were exposed to different concentrations of CSE (2.5, 5%) for different lengths of time (2, 6 hours), with or without co-treatment with Puerarin and/or TEMPO. Cells were exposed to Puerarin (20, 40 *μ*M) for 6 hours prior to CSE stimulation. Alternatively, cells were co-treated with TEMPO (100 *μ*M) with or without Puerarin (40 *μ*M) for 1 hour prior to CSE stimulation. The doses of Puerarin [23] and TEMPO [21] used for HSAEC treatments were selected based on existing literatures documenting selective effects of these agents on cell signaling without inducing cytotoxicity.

### 2.9. Preparation of Cigarette Smoke Extract

Cigarette smoke extract (CSE) solution was prepared as previously described with modifications [24]. Briefly, one cigarette was smoked continuously by the apparatus described in the previous study [24]. Cigarette smoke was drawn through 10 mL of DMEM/F12 (Lonza Biosicence, Walkersville, MD, USA) by application of a vacuum to a 50 mL centrifuge tube containing DMEM/F12 medium. Each cigarette was smoked approximately for 5 min, and the smoke dissolved in 10 mL DMEM/F12 generated a 100% CSE stock solution. CSE solution was filtered through a 0.22 *μ*m syringe filter and standardized by measuring the absorbance (OD = 0.8 ± 0.05) at a wavelength of 320 nm as previously reported [25]. A 100% CSE stock corresponds to one cigarette/10 mL medium in this system. As the average blood volume of a person is approximately 5 L, 5% CSE preparation is equivalent to 25 cigarettes/person. Hence, 2.5 and 5% CSE concentration were used in the present studies for mimicking the average intake of a smoker who smokes half a pack (10 cigarettes) or one pack (20 cigarettes) per day.

### 2.10. Immunohistochemistry and Immunocytochemistry Analyses of Inflammatory Meditor Expression and p65 Nuclear Translocation

Freshly isolated mouse lungs were perfused, formalin fixed, paraffin embedded, and sectioned (5 *μ*m). Longitudinal sections of the left lung were rehydrated through a series of xylene (2×) and ethanol gradient (2× absolute, 90%, 80%, 70%, 50%), and antigen retrieval with EDTA-TRIS (10 mM citric acid and 0.05% Tween 20, pH 9.0) at 95°C for 30 min. Sections were then blocked with 0.3% H_2_O_2_ for 30 min, washed with PBS (3 min each) and incubated with phospho-p65 (Ser536) antibody (1 : 500 dilution, Cell Signaling Technology, Danvers, MA, USA), COX-2 antibody (1 : 100, Cayman Chemical, Ann Arbor, MI, USA), IL-6 antibody (1 : 400, Abcam, Boston, MA, USA), or MCP-1 antibody (1 : 500, NOVUS biologicals, Littleton, CO, USA) followed by incubation with secondary antibodies (Dako Corp, Carpinteria, CA, USA) conjugated to horseradish peroxidase, and finally imaged using 3,3′-diaminobenzidine chromogenic-substrate (DAB) substrate (Dako Corp, Carpinteria, CA, USA) according to manufacturer's instructions. Sections were counterstained with hematoxylin, mounted, and imaged using a BX51 fluorescent microscope (Olympus, Tokyo, Japan). The images were quantified using the NIH ImageJ software. The mean optical density (IOD) of positive staining was calculated by measuring 5-8 consecutive visual fields for each sample at a magnification of ×400.

To evaluate NF-*κ*B nuclear translocalization in CSE exposed HSAECs, immunofluorescent staining of the (NF-*κ*B) p65 subunit was performed. HSAECs grown on eight chamber slides were stained using rabbit NF-*κ*B p65 antibody (1 : 500, Cell Signaling Technology, Danvers, MA, USA) and Alexa Fluor-555 conjugated secondary antibody (1 : 500, Abcam, Boston, MA, USA). The images were captured using Nikon A1 + R Confocal Microscope (Tokyo, Japan) with excitation and emission wavelengths of 488 and 585 nm, respectively.

### 2.11. Enzyme-Linked Immunosorbant Assay (ELISA) for Detection of IL-8 Release

HSAECs were cultured in 24 well plates until 80-90% confluence and treated with 5% CSE with or without Puerarin pre-treatment (40 *μ*M, 6 hours). After 24 hours, the culture medium was collected and centrifuged at 2,500 rpm for 5 min to pellet cells. The supernatants were diluted 30-fold prior to ELISA determination of IL-8 levels using the IL-8/CXCL8 Valukine ELISA kit (Novus Biologicals, Littleton, CO, USA). The supernatants were analyzed in duplicate, and absorbance was measured at 450/540 nm using a BioTek Plate Reader (SYNERGY-HTX, Molecular Devices, Sunnyvale, CA, USA).

### 2.12. Western Blotting Analyses of NOX Isoforms and Inflammatory Mediators

Lung tissue lysates and whole-cell lysates were prepared using protein lysis buffer (20 mM Tris-HCL, 150 mM Nacl, 1 mM EDTA, 1 mM EGTA, 2.5 mM sodium pyrophosphate, 1.22 mM MgSO4, 20 mM Tris-HCL, 150 mM Nacl) containing protease inhibitor cocktail (MilliporeSigma, Louis, MO, USA). The total protein concentration was determined using a BCA Protein Assay Kit (Cell Signaling Technology, Danvers, MA, USA). Equal amounts of protein (30-50 *μ*g) were separated by 10% SDS-PAGE and then transferred onto polyvinylidene fluoride (PVDF) membranes. The membrane was blocked with 5% w/v BSA at room temperature for 1 hour and subsequently incubated with antibodies for NOX1 (1 : 1000 dilution, Novus Biologicals, Littleton, CO, USA), NOX2 (gp91phox) (1 : 100, Santa Cruz Biotechnology, Dallas, Texas, USA), NOX4 (1 : 500, Novus Biologicals, Littleton, CO, USA), NOX5 (1 : 500, Novus Biologicals, Littleton, CO, USA), COX-2 (1 : 200, Cayman Chemical, Ann Arbor, MI, USA), TNF-*α* (1 : 500, Abcam, Boston, MA, USA), GAPDH (1 : 10,000, Abcam, Boston, MA, USA) and *β*-actin (1 : 5,000, Abcam, Boston, MA, USA) overnight in 5% w/v BSA at 4°C with gentle shaking. Then, the membranes were incubated with secondary antibodies at room temperature with gentle shaking for 1 hour. The bands were visualized using electrochemiluminescence reagents (MilliporeSigma, Louis, MO, USA) and the Gel Doc™ XR + System (Bio-Rad Laboratories, Hercules, CA, USA), and quantified using the NIH ImageJ software.

### 2.13. Statistical Analysis

All data are presented as mean ± SEM from three to six independent experiments. Statistical analyses were performed using Graphpad Software. Student's *t*-test was used for two group comparisons, and ANOVA was used to compare means of multiple experimental groups, followed by Tukey's posthoc test. The significant difference was set as *p* < 0.05.

## 3. Results

### 3.1. Purerarin Attenuated ACS-Induced Increase in Superoxide Production and Upregulation of NOX Isoforms in Mice

**Figure 1:**
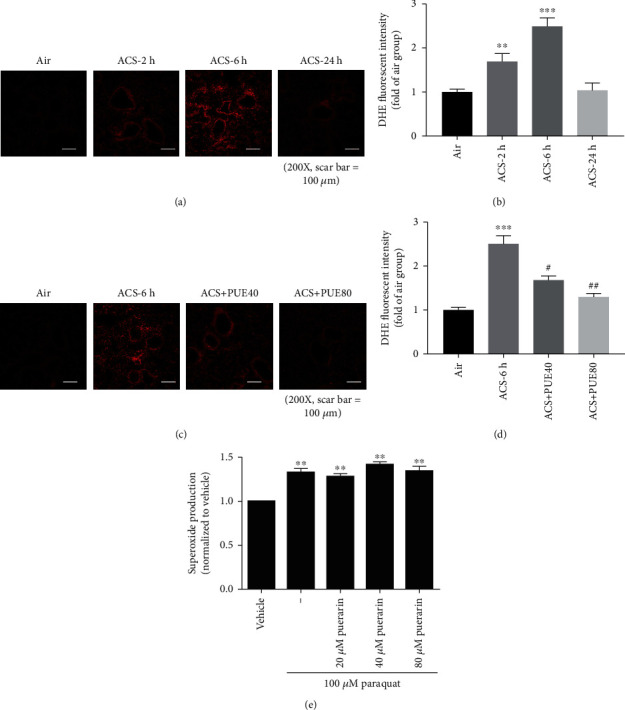
Puerarin attenuated ACS-induced superoxide production in mice. Mice were exposed to whole body cigarette smoking for 1 hour and lung tissues harvested at different time points (2, 6 and 24 hours) for DHE fluorescent imaging of superoxide production. Puerarin (40 or 80 mg/kg) was intraperitoneally injected into mice for 1 hour prior to and after ACS and then lung tissues harvested at 6 hours. Freshly isolated lung tissues were embedded in OCT, frozen at -20°C and sectioned immediately, and then sections incubated with DHE (5 *μ*M) for 30 min in the dark. After washing, the sections were mounted and imaged by Nikon A1+ confocal microscope. The images were quantified using NIH ImageJ software. (a, b) Representative DHE images and quantitative data of superoxide production at different time points after ACS exposure in mice. Data are shown as mean ± SEM, ^∗∗^*p* < 0.01, ^∗∗∗^*p* < 0.001 vs. control/air group. *n* = 4 − 9. (c, d) Representative images and quantitative data of superoxide production in ACS exposed mice with or without Puerarin treatment. Data are shown as mean ± SEM. ^∗∗∗^*p* < 0.001 vs. control/air group; ^#^*p* < 0.05, ^##^*p* < 0.01 vs. ACS group. *n* = 4 − 9. (e) Effect of Puerarin on paraquat generated superoxide in a cell-free buffer system. Superoxide production was determined by electron spin resonance (ESR) as previously published. Paraquat (100 *μ*M) generated superoxide in a cell-free buffer system, which was not changed by Puerarin at indicated concentrations. These data indicate that Puerarin does not scavenge superoxide by direct chemical reaction. Data are shown as mean ± SEM, ^∗∗^*p* < 0.01 vs. control/vehicle group. *n* = 3. PUE40: Puerarin (40 mg/kg); PUE80: Puerarin (80 mg/kg).

Oxidative stress is a major driving mechanism during the pathogenesis of COPD, which activates inflammatory responses of the airways and parenchyma [10]. To examine superoxide production in response to acute cigarette smoking (ACS), mice were exposed to whole body cigarette smoking for 1 hour, and the left lung was harvested for DHE fluorescent imaging analysis of superoxide production. One hour exposure to ACS significantly increased superoxide production in mouse lung tissues, shown by representative DHE images and quantitative data, detected at 2, 6, and 24 hours post ACS exposure (Figures 1(a) and 1(b)). Intriguingly, Puerarin dose-dependently abrogated ACS-induced superoxide production (Figures 1(c) and 1(d)).

Next, we examined Puerarin's effects on directly scavenging superoxide by electron spin resonance (ESR) spectrophotometer to examine whether the inhibitory effects of Puerarin on superoxide production are related to direct chemical scavenging activity of Puerarin on superoxide anion. Paraquat generation of superoxide radicals in a buffer system was used to examine whether addition of Puerarin diminishes superoxide signal detected by ESR. As is clear in Figure 1(e), Puerarin had no effect in directly buffering superoxide in a cell free system. Therefore, we further investigated molecular pathways mediating Puerarin's beneficial effects on attenuating ROS production.

**Figure 2:**
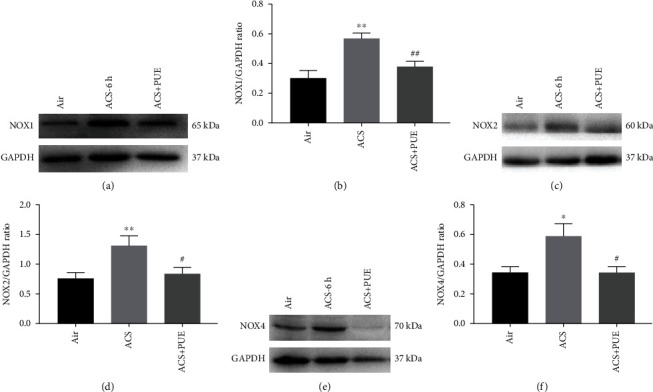
Puerarin attenuated ACS-induced upregulation of NOX isoforms in mice. Mice were exposed to whole body cigarette smoking for 1 hour, and Puerarin (80 mg/kg) was intraperitoneally injected into mice for 1 hour prior to and after ACS. Lung tissues were harvested at 6 hours after ACS and lysed to examine protein expression of NOX1, NOX2, or NOX4 by Western blotting analyses. The bands were visualized using the Gel Doc™ XR + System and intensity quantified using NIH ImageJ software. (a, b) Representative Western blots and grouped data of NOX1 protein expression in ACS exposed mice with and without Puerarin treatment. Data are shown as mean ± SEM. ^∗∗^*p* < 0.01 vs. control/air group; ^##^*p* < 0.01 vs. ACS group. *n* = 3 − 5. (c, d) Representative Western blots and grouped data of NOX2 protein expression in ACS exposed mice with and without Puerarin treatment. Data are shown as mean ± SEM. ^∗∗^*p* < 0.01 vs. control/air group; ^#^*p* < 0.05 vs. ACS group. *n* = 5 − 6. (e-f) Representative Western blots and grouped data of NOX4 protein expression in ACS exposed mice with and without Puerarin treatment. Data are shown as mean ± SEM. ^∗^*p* < 0.05 vs. control/air group; ^#^*p* < 0.05 vs. ACS group. *n* = 4 − 6. PUE: Puerarin.

We examined roles of NOX isoforms in mediating ROS production in response to ACS exposure in mice and the effects of Puerarin on NOX activation. Since our data indicate that high concentrations of Puerarin (80 mg/kg) had more profound effects on superoxide production, we used this concentration of Puerarin for subsequent studies to examine effects of Puerarin on NOX isoforms in ACS exposed mice. Data indicate that protein expression of NOX1, NOX2, or NOX4 was upregulated in ACS exposed mice, and that Puerarin treatment (80 mg/kg) completely or markedly attenuated this upregulation in NOX isoforms in mice at 6 hours after ACS exposure. Shown in Figure 2 are representative Western blots and grouped data of NOX isoform protein expression in ACS exposed mice with or without Puerarin administration (Figures 2(a)–2(f)).

### 3.2. Puerarin Attenuated ACS-Induced Inflammatory Cell Recruitment in Lung Parenchyma and BAL Fluid

**Figure 3:**
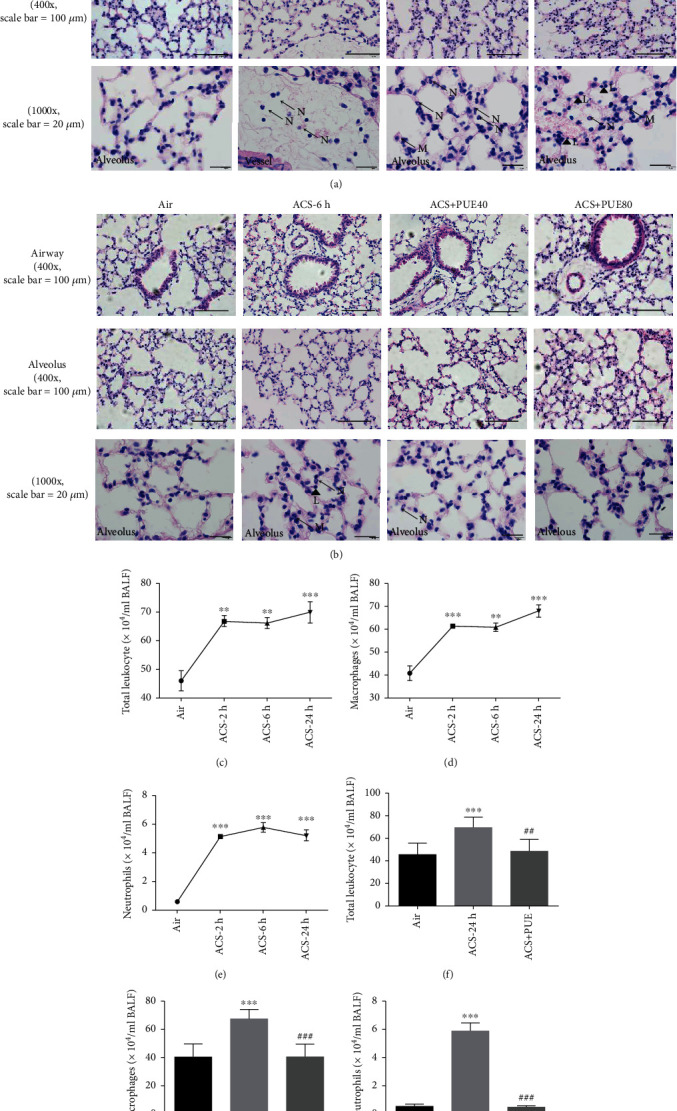
Puerarin attenuated ACS-induced recruitments of inflammatory cells in lung parenchyma and BAL fluid. Mice were exposed to whole body cigarette smoking for 1 hour and lung tissues harvested at different time points (2, 6, and 24 hours) for H&E imaging analysis. In parallel experiments, Puerarin (40 or 80 mg/kg) was intraperitoneally injected into mice for 1 hour prior to and after ACS and then lung tissues harvested at 6 hours for H&E imaging analysis. Besides, bronchoalveolar lavage (BAL) fluid was collected at 2, 6, and 24 hours after ACS or at 6 hrs in mice with or without treatment with Puerarin (80 mg/kg), before staining with Giemsa for inflammatory cell enumerating. H&E and Giemsa images were captured using a BX51 fluorescent microscope. (a, b) Representative H&E images of lung sections at different time points after ACS exposure in mice and in lung sections of ACS exposed mice with or without Puerarin treatment. Shown form top panel to bottom panel are images of airway, alveolus, and inflammatory cells in sequence. The arrows indicate neutrophils (N) or macrophages (M) while the triangles indicate lymphocytes (L). The magnification is ×400 for the upper two rows while the magnification is 1000x for the bottom row. (c)–(e) Numbers of total leukocytes (c), macrophages (d), and neutrophils (e) in BAL fluid of ACS exposed mice at different time points. (f)–(h) Numbers of total leukocytes (f), macrophages (g), and neutrophils (h) in BAL fluid of ACS exposed mice with or without Puerarin (80 mg/kg) treatment. Data are shown as mean ± SEM. ^∗∗^*p* < 0.01, ^∗∗∗^*p* < 0.001 vs. control/air group; ^##^*p* < 0.01, ^###^*p* < 0.001 vs. ACS group. *n* = 3 − 8. PUE: Puerarin.

We next examined ACS-induced inflammatory responses by assessing types of inflammatory cells accumulated in H&E stained lung sections. We found that ACS exposure resulted in perivascular inflammation with extravasating neutrophils appearing 2 hours after ACS. Six and 24 hours after ACS, the accumulation of macrophages and neutrophils was obvious in alveolar capillaries, and lymphocytes were found in bronchoalveolar space 24 hours after ACS. In the presence of Puerarin treatment (80 mg/kg), the recruitments of neutrophils and macrophages were completely attenuated in lung tissue sections compared to the ACS group (Figures 3(a) and 3(b)).

Previous study has shown that the total number of neutrophils and macrophages increases in BAL fluid at 24 hours after ACS exposure [26]. Indeed, the total number of cells in BAL fluid of ACS exposed mice was time-dependently increased comparing to the control group, especially at 24 hours after ACS exposure (70.00 ± 3.71 × 10^4^ cells/mL vs. 46.09 ± 3.52 × 10^4^ cells/mL for ACS 24 hr group vs. control/air group; Figure 3(c)). The predominant type of cells in BALF of ACS exposed mice was macrophages, number of which was also time-dependent increased in BAL fluid following ACS exposure (Figure 3(d)). The number of neutrophils was also increased in ACS groups at different time points (Figure 3(e)). Importantly, Puerarin treatment at 80 mg/kg completely attenuated increases in the total number of inflammatory cells and the numbers of macrophages and neutrophils in BAL fluid, comparing to the untreated ACS group at 24 hours (Figures 3(f)–3(h)).

### 3.3. Puerarin Attenuated ACS-Induced Upregulation of TNF-*α* and KC mRNA Expression and Activation of NF-*κ*B Pathway in Mice

**Figure 4:**
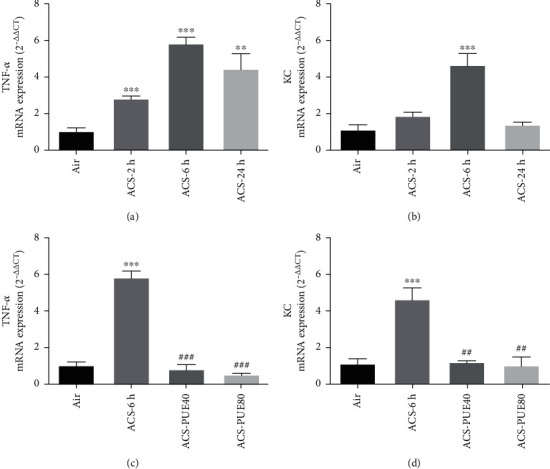
Puerarin attenuated ACS-induced upregulation of TNF-*α* and KC mRNA expression in mice. Mice were exposed to whole body cigarette smoking for 1 hour and lung tissues harvested at different time points (2, 6, and 24 hours) for RT-PCR analysis of TNF-*α* and KC mRNA expression with *β*-actin as an endogenous control. In parallel experiments, Puerarin (40 or 80 mg/kg) was intraperitoneally injected into mice for 1 hour prior to and after ACS and then lung tissues harvested at 6 hours for RT-PCR analysis of TNF-*α* and KC mRNA expression. Shown are mRNA expression levels of TNF-*α* (a) and keratinocyte chemoattractant (KC) (b) in ACS exposed mice at different time points. (c, d) mRNA expression levels of TNF-*α* (c) and KC (d) in ACS exposed mice after 6 hours with or without Puerarin treatment. Data are shown as mean ± SEM. ^∗∗^*p* < 0.01, ^∗∗∗^*p* < 0.001 vs. control/air group; ^##^*p* < 0.01, ^###^*p* < 0.001 vs ACS group. *n* = 3 − 5. PUE40: Puerarin (40 mg/kg); PUE80: Puerarin (80 mg/kg).

Given that ACS exposed mice had increased numbers of inflammatory cells in lung tissue and BAL fluid, we next assessed gene expression of proinflammatory cytokines in lung tissues by RT-PCR. The mRNA expression of TNF-*α* and KC (murine homolog IL-8) in the lung tissues was significantly upregulated by almost 3-fold compared to the control/air group, especially at 6 hours after ACS exposure (Figures 4(a) and 4(b)). These responses were significantly attenuated by Puerarin treatment (40 or 80 mg/kg) in a dose-dependent manner (Figures 4(c) and 4(d)).

**Figure 5:**
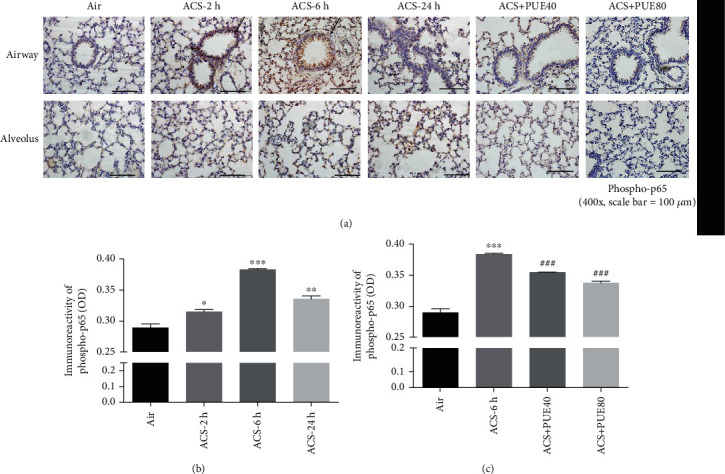
Puerarin attenuated ACS-induced NF-*κ*B activation in mice. Mice were exposed to whole body cigarette smoking for 1 hour and lung tissue harvested at different time points (2, 6, and 24 hours), and then the left lung was paraffin-embedded for immunohistochemical staining of phospho-p65. In parallel experiments, Puerarin (40 or 80 mg/kg) was intraperitoneally injected into mice for 1 hour prior and after ACS and then lung tissues harvested at 6 hours for immunohistochemical staining of phospho-p65. Positive immunoreactivity was visualized using 3,39-diaminobenzidine (DAB) substrate (brown). Sections were counterstained with Mayer's hematoxylin (blue) and imaged using a BX51 fluorescent microscope. The images were quantified using NIH ImageJ software. (a) Representative immunohistochemical images of phospho-p65 staining on airway epithelium (upper panels) and alveolus (lower panels) in ACS exposed mice with or without Puerarin treatment. (b, c) Quantified data of phospho-p65 in ACS exposed mice with or without Puerarin treatment. Data are shown as mean ± SEM. ^∗^*p* < 0.05, ^∗∗^*p* < 0.01^∗∗∗^*p* < 0.001 vs. control/air group. ^###^*p* < 0.001 vs. ACS group. *n* = 3 − 5. PUE40: Puerarin (40 mg/kg); PUE80: Puerarin (80 mg/kg).

NF-*κ*B is a key transcription factor regulating many proinflammatory genes in COPD [10], and known to play an important role in inflammatory responses in ACS exposed mice [27]. We examined expression of phospho-(NF-кB) p65 in ACS exposed mouse lungs by immunohistochemistry. The expression of phospho-p65 was significantly increased in ACS group compared to the control group, especially at 6 hours after ACS exposure (Figures 5(a)–5(c)). Phospho-p65 was mainly upregulated in airway epithelium and alveolar macrophages (Figures 5(a) and 5(b)). Interestingly, expression of phospho-p65 significantly increased in airway epithelium at 2 and 6 hours after ACS exposure in mice, while it was increased in alveoli at 6 and 24 hours after ACS exposure. Quantitatively, Puerarin treatment at 40 or 80 mg/kg significantly attenuated ACS-induced phospho-p65 expression in a dose-dependent manner (Figure 5(c)).

### 3.4. Puerarin Attenuated ACS-Induced Protein Expression of TNF-*α*, COX-2, IL-6, and MCP-1 in Mice

**Figure 6:**
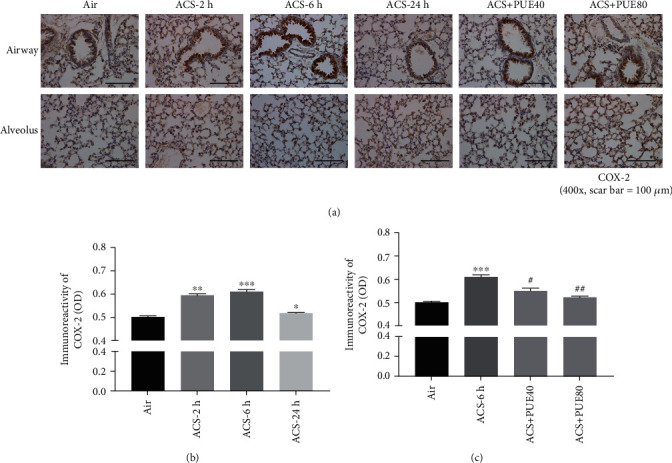
Puerarin attenuated ACS-induced COX-2 upregulation in mice. Mice were exposed to whole body cigarette smoking for 1 hour and lung tissue harvested at different time points (2, 6, and 24 hours), and then the left lung was paraffin-embedded for immunohistochemical staining of COX-2 expression. In parallel experiments, Puerarin (40 or 80 mg/kg) was intraperitoneally injected into mice for 1 hour prior to and after ACS exposure and lung tissues harvested at 6 hours for immunohistochemical staining of COX-2 expression. Positive immunoreactivity was visualized using 3,39-diaminobenzidine (DAB) substrate (brown). Sections were counterstained with Mayer's hematoxylin (blue) and imaged using a BX51 fluorescent microscope. The images were quantified using NIH ImageJ software. (a) Representative immunohistochemical images of COX-2 staining on airway epithelium (upper panels) and alveolus (lower panels) in ACS exposed mice with or without Puerarin treatment. (b, c) Quantitative data of COX-2 expression in ACS exposed mice with or without Puerarin treatment. Data are shown as mean ± SEM. ^∗^*p* < 0.05, ^∗∗^*p* < 0.01^∗∗∗^*p* < 0.001 vs. control/air group; ^#^*p* < 0.05, ^##^*p* < 0.01 vs. ACS group. *n* = 3 − 6. PUE40: Puerarin (40 mg/kg); PUE80: Puerarin (80 mg/kg).

As COX-2 promoter is subjected to tight regulation involving NF-кB [28], we examined protein expression of COX-2 by immunohistochemistry in ACS exposed mouse lungs. Data indicate that ACS significantly increased protein expression of COX-2 in airway epithelium, most prominently at 6 hours post ACS exposure (Figures 6(a) and 6(b)). Puerarin treatment (40 or 80 mg/kg) markedly attenuated protein expression of COX-2 in a dose-dependent manner (Figure 6(c)).

**Figure 7:**
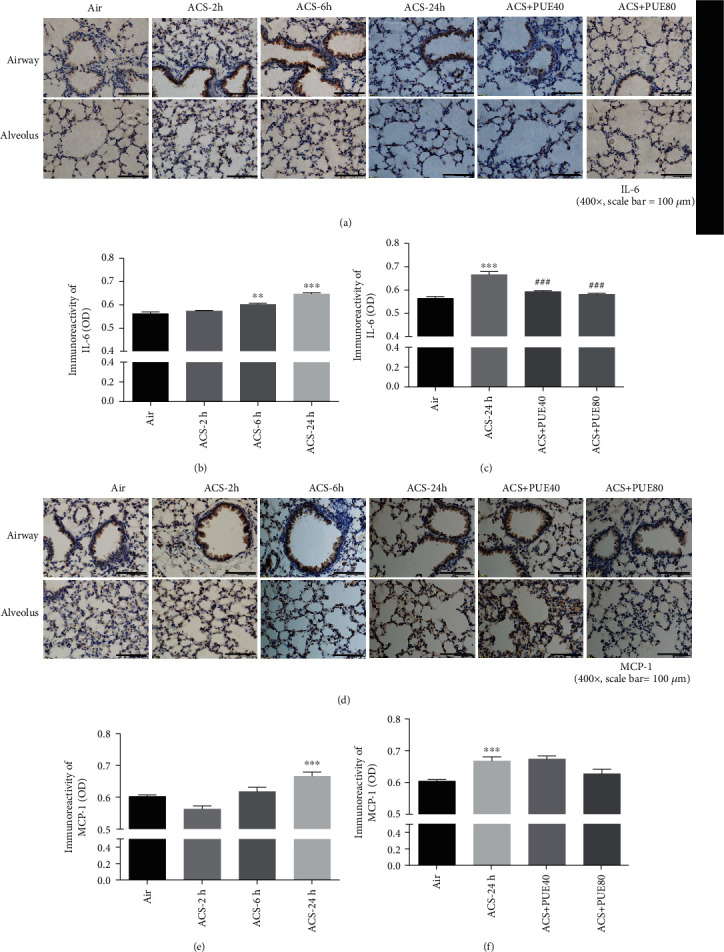
Puerarin attenuated ACS-induced IL-6 and MCP-1 expression in mice. Mice were exposed to whole body cigarette smoking for 1 hour and lung tissue harvested at different time points (2, 6, and 24 hours), and then the left lung was paraffin-embedded for immunohistochemical staining of IL-6 and MCP-1 expression. In parallel experiments, Puerarin (40 or 80 mg/kg) was intraperitoneally injected into mice for 1 hour prior to and after ACS exposure and then lung tissues harvested at 24 hours for immunohistochemical staining of IL-6 and MCP-1 expression. Positive immunoreactivity was visualized using 3,39-diaminobenzidine (DAB) substrate (brown). Sections were counterstained with Mayer's hematoxylin (blue) and imaged using a BX51 fluorescent microscope. The images were quantified using NIH ImageJ software. (a, d) Representative immunohistochemical images of IL-6/MCP-1 on airway epithelium (upper panels) and alveoli (lower panels) in ACS exposed mice with or without Puerarin treatment. (b, c) Grouped data of IL-6 expression in ACS exposed mice with or without Puerarin treatment. Data are shown as mean ± SEM. ^∗∗^*p* < 0.01, ^∗∗∗^*p* < 0.001 vs. control/air group; ###*p* < 0.001 vs. ACS group. *n* = 3 − 5. (e, f) Grouped data of MCP-1 expression in ACS exposed mice with or without Puerarin treatment. Data are shown as mean ± SEM. ^∗∗∗^*p* < 0.001 vs. control/air group. *n* = 3 − 6. PUE40: Puerarin (40 mg/kg); PUE80: Puerarin (80 mg/kg).

IL-6 and MCP-1 are essential proinflammatory cytokines that have been implicated in COPD. We examined expression of IL-6 and MCP-1 in ACS exposed mouse lungs by immunohistochemistry. Data indicate that ACS significantly increased protein expression of IL-6 in airway epithelium at 6 and 24 hours after ACS exposure, most prominently at 24 hours (Figures 7(a) and 7(b)). Puerarin treatment abrogated upregulation of IL-6 in a dose-dependent manner (Figures 7(a) and 7(c)). The protein expression of MCP-1 was significantly increased in ACS exposed mouse lungs compared to the control group, especially at 24 hours after ACS exposure (Figures 7(d) and 7(e)). Quantitatively, Puerarin treatment at 40 or 80 mg/kg attenuated ACS-induced MCP-1 expression, at marginal statistical significance (Figures 7(d) and 7(f)).

**Figure 8:**
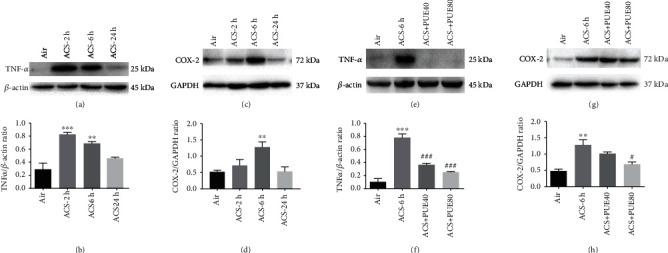
Puerarin attenuated ACS-induced protein expression of TNF-*α* and COX-2. Mice were exposed to whole body cigarette smoke for 1 hour and lung tissue harvested at different time points (2, 6, and 24 hours), and then the right lung was harvested for Western blotting analyses of TNF-*α* and COX-2 protein expression. In parallel experiments, Puerarin (40 or 80 mg/kg) was intraperitoneally injected into mice for 1 hour prior to and after ACS and then lung tissues harvested at 6 hours for Western blotting analyses of TNF*α* and COX-2 protein expression. (a, c) Representative Western blots of TNF-*α* and COX-2 expression in ACS exposed mouse lungs at different time points. (b, d) Grouped densitometric data of TNF-*α* and COX-2 in ACS exposed mouse lungs at different time points. Data are shown as mean ± SEM. ^∗∗^*p* < 0.01, ^∗∗∗^*p* < 0.001 vs. control/air group. *n* = 3 − 6. (e, g) Representative Western blots of TNF-*α* and COX-2 expression in ACS exposed mouse lungs with or without Puerarin treatment. (f, h) Grouped densitometric data of TNF-*α* and COX-2 in ACS exposed mouse lungs with or without Puerarin treatment. Data are shown as mean ± SEM. ^∗∗^*p* < 0.01, ^∗∗∗^*p* < 0.001 vs. control/air group; ^#^*p* < 0.05, ^###^*p* < 0.001. *n* = 3 − 5. PUE40: Puerarin (40 mg/kg); PUE80: Puerarin (80 mg/kg).

Protein expression of TNF-*α* and COX-2 was further evaluated using Western blotting analyses. Data indicate that TNF-*α* protein expression was significantly increased at 2 hours and 6 hours after ACS exposure (Figure 8(a); *p* < 0.05), and COX-2 expression was also significantly upregulated 6 hours after ACS exposure (Figure 8(b)). Quantitatively, Puerarin treatment at 40 or 80 mg/kg markedly attenuated upregulation of TNF-*α* and COX-2 expression in a dose-dependent manner (Figures 8(e) and 8(f)). Taken together, these data demonstrate that ACS-induced inflammatory cell recruitments, activation of NF-*κ*B pathway, and upregulation of inflammatory meditors of TNF-*α*, COX-2, IL-6 and MCP-1 were all effectively alleviated by Puerarin treatment in mice.

### 3.5. Puerarin Suppressed CSE Stimulated ROS Production and Upregulation of NOX Isoforms in HSAECs

**Figure 9:**
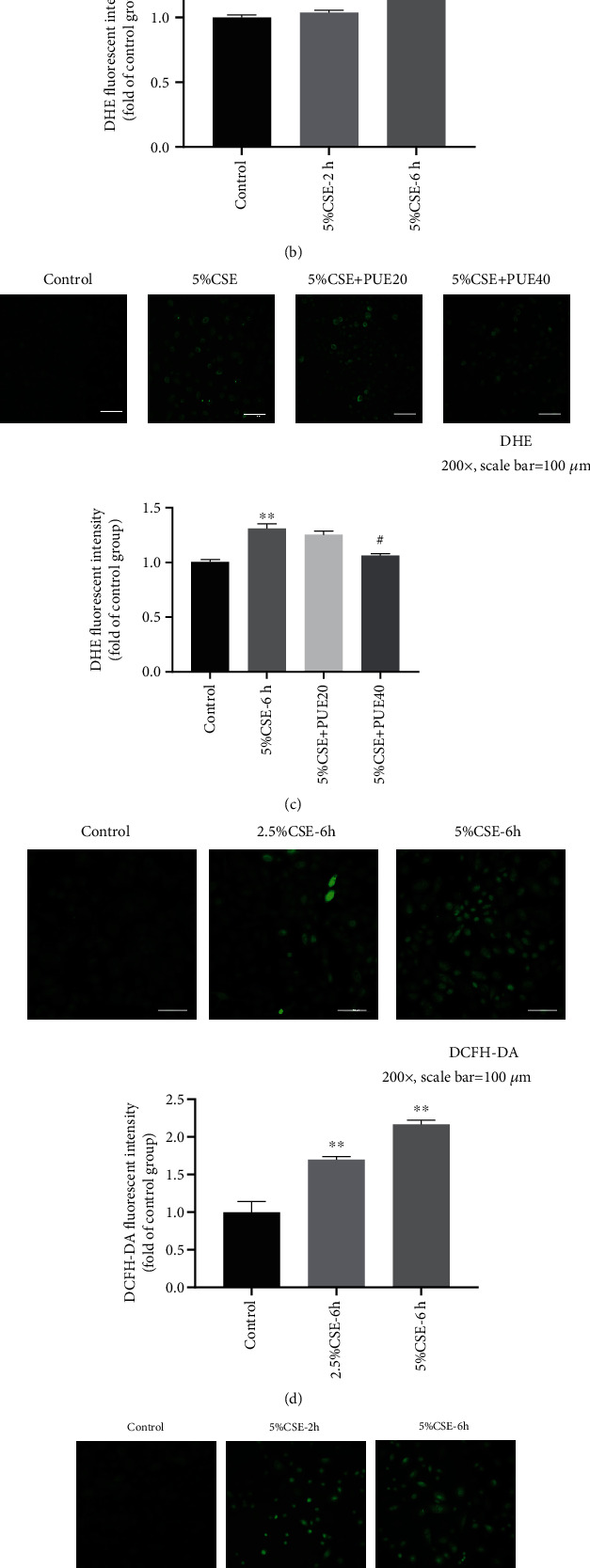
Puerarin suppressed CSE-induced ROS production in HSAECs. Human small airway epithelial cells (HSAECs) cultured in 24 well plates were incubated with 2.5% or 5% cigarette smoke extract (CSE) for 2 hr or 6 hr. Besides, HSAECs were stimulated with 5% CSE for 6 hr with or without Puerarin treatment (20 *μ*M or 40 *μ*M). Superoxide or hydrogen peroxide production in CSE-induced HSAECs was detected by DHE (2 *μ*M) or DCFH-DA (20 *μ*M) fluorescent imaging, respectively. After washing, HSAECs were mounted and imaged by Nikon A1+ confocal microscope. The DHE and DCFH-DA images were quantified using NIH ImageJ software. Shown are dose-dependent (a) and time-dependent (b) superoxide production in CSE stimulated HSAECs. (a) Representative DHE images and quantitative data of superoxide production in HSAECs at 6 hours following 2.5% or 5% CSE stimulation. (b) Representative DHE images and quantitative data of superoxide production in 5% CSE stimulated HSAECs at 2 or 6 hours. (c) Representative DHE images and quantitative data of superoxide production in HSAECs at 6 hours following CSE stimulation with or without Puerarin treatment (20 *μ*M or 40 *μ*M) (magnified ×200, scale bar = 100 *μ*m). Data are shown as mean ± SEM. ^∗∗^*p* < 0.01 vs. control/air group. ^#^*p* < 0.05. and ^##^*p* < 0.01 vs. CSE group, *n* = 3 − 4. Shown are dose-dependent (d) and time-dependent (e) hydrogen peroxide production in CSE stimulated HSAECs. (d) Representative DCFH-DA images and quantitative data of hydrogen peroxide production in HSAECs at 6 hours following 2.5% or 5% CSE stimulation. (e) Representative DCFH-DA images and quantitative data of hydrogen production in 5% CSE stimulated HSAECs at 2 or 6 hours. (f) Representative DCFH-DA images and quantitative data of hydrogen peroxide production in HSAECs at 6 hours following CSE stimulation with or without Puerarin treatment (20 *μ*M or 40 *μ*M) (magnified ×200, scale bar = 100 *μ*m). Data are shown as mean ± SEM. ^∗∗^*p* < 0.01 vs. control/air group. ^#^*p* < 0.05 and ^##^*p* < 0.01 vs. CSE group, *n* = 3 − 4. PUE20: Puerarin (20 *μ*M); PUE40: Puerarin (40 *μ*M).

As CSE stimulated HSAECs represent a recognized model system in vitro for mechanistic studies of COPD, we assessed CSE induced ROS production in HSAECs and the anti-oxidative and anti-inflammatory effects of Puerarin. CSE was freshly prepared as described in the Methods section and immediately added to the cultural medium of HSAECs. The intracellular superoxide and hydrogen peroxide production in HSAECs was measured by DHE and DCFH-DA fluorescent imaging, respectively. Of note, CSE induced time and dose-dependent increases in intracellular ROS production (Figure 9). Importantly, CSE induced increases in intracellular ROS production were markedly attenuated by Puerarin in a dose-dependent manner (Figure 9). These data indicate that Puerarin can effectively abrogate CSE induced ROS production in HSAECs.

**Figure 10:**
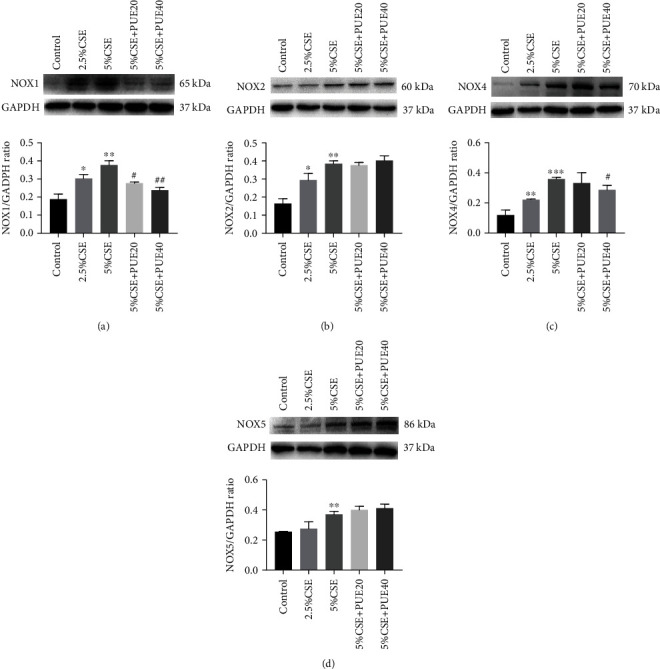
Puerarin attenuated protein expression of NOX1 and NOX4 in CSE stimulated HSAECs. Human small airway epithelial cells (HSAECs) cultured in 6-well plates were stimulated with CSE (2.5% and 5%) for 6 hours with or without treatment with Puerarin (20 *μ*M and 40 *μ*M), and then lysed to examine protein expression of NOX1, NOX2, NOX4, or NOX5 by Western blotting analyses. (a)–(d) Representative Western blots and grouped densitometric data of NOX1 (a), NOX2 (b), NOX4 (c), and NOX5 (d) protein expression in CSE stimulated HSAECs with or without Puerarin treatment. Data are shown as mean ± SEM. ^∗^*p* < 0.05, ^∗∗^*p* < 0.01, ^∗∗∗^*p* < 0.001 vs. control group; ^#^*p* < 0.05, ^##^*p* < 0.01 vs. CSE group. *n* = 3 − 5. PUE20: Puerarin (20 *μ*M); PUE40: Puerarin (40 *μ*M).

Next, we examined protein expression of NOX isoforms in CSE stimulated HSAECs by Western blotting analyses. Data indicate that protein expression of NOX1 and NOX4 was upregulated in CSE stimulated HSAECs, which was significantly abrogated by Puerarin treatment (20 *μ*M or 40 *μ*M) in a dose-dependent manner (Figures 10(a)–10(d)). In contrast, protein expression of NOX2 and NOX5 was also upregulated by CSE stimulation but unaffected by Puerarin.

### 3.6. Puerarin Attenuated p65 Nuclear Translocation, COX-2 Protein Expression, and IL-8 Release in CSE Stimulated HSAECs

**Figure 11:**
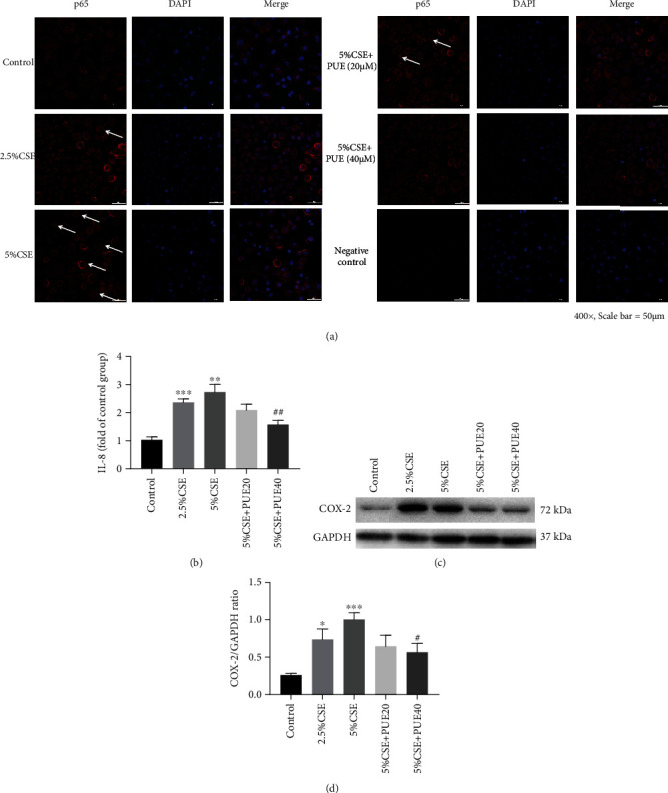
Puerarin attenuated p65 translocation, COX-2 expression, and IL-8 release in CSE stimulated HSAECs. Human small airway epithelial cells (HSAECs) cultured on slides in 8-well chambers were stimulated with CSE (2.5% and 5%) for 30 min with or without treatment with Puerarin (20 *μ*M and 40 *μ*M). The cells were incubated with NF-*κ*B RelA/p65 antibody (1 : 500) for immunofluorescent assay, and the images were captured by Nikon A1 + R Confocal microscope. (a) Shown is nuclear translocation of RelA/p65 (red) in CSE stimulated HSAECs. The arrows indicate nuclear translocation of RelA/p65 (×400, scale bar = 50 um) that was dose-dependently increased by CSE exposure and robustly attenuated by Puerarin treatment. In parallel experiments, HSAECs cultured in 96-well plates were stimulated with CSE (2.5% and 5%) for 24 hours with or without treatment with Puerarin (20 *μ*M and 40 *μ*M) and then cell culture medium collected for measuring IL-8 levels by ELISA in duplicate using BioTek Plate Reader. (b) Release of IL-8 in culture medium of CSE stimulated HSAECs with or without treatment with Puerarin (20 *μ*M and 40 *μ*M). Data are shown as mean ± SEM. ^∗∗∗^*p* < 0.001, ^∗∗^*p* < 0.01 compared to the control group. ^##^*p* < 0.01 compared with the CSE group, *n* = 3 − 5. Additionally, HSAECs cultured in 6-well plate were stimulated with CSE (2.5% and 5%) for 6 hours with or without treatment with Puerarin (20 *μ*M and 40 *μ*M) and then lysed to examine protein expression of COX-2 by Western blotting analysis. (c, d) Representative Western blots and grouped densitometric data of COX-2 protein expression CSE stimulated HSAECs with or without Puerarin treatment. Data are shown as mean ± SEM. ^∗^*p* < 0.05, ^∗∗∗^*p* < 0.001 vs. control group. ^#^*p* < 0.05 vs. CSE group, *n* = 3 − 5. PUE20: Puerarin (20 *μ*M); PUE40: Puerarin (40 *μ*M).

We further examined effects of Puerarin on inflammatory responses in CSE stimulated HSAECs. Since nuclear translocation of p65 indicates activation of NF-*κ*B, we examined translocation of NF-*κ*B (p65) in CSE stimulated HSAECs by immunocytochemistry. Of note, NF-*κ*B (p65) was translocated to the nuclei in CSE stimulated HSAECs at 30 min, which was significantly reversed by Puerarin administration (Figure 11(a)). IL-8 is an important inflammatory cytokine implicated in the pathogenesis of COPD, which was found increased in the supernatant of CSE stimulated HSAECs [25]. We next measured levels of IL-8 in the culture medium of CSE treated HSAECs by ELISA. Cells treated with 5% CSE had significantly increased release of IL-8 in culture media at 24 hours, and this response was attenuated by Puerarin in a dose dependent manner (Figure 11(b)). Further, protein expression of COX-2 was determined by Western blotting analysis. As shown in Figures 11(c) and 11(d), COX-2 protein abundance was significantly increased in CSE stimulated HSAECs 6 hours post CSE exposure. This response was significantly attenuated by Puerarin treatment (20 *μ*M or 40 *μ*M) in a dose-dependent manner (Figures 11(c) and 11(d); *p* < 0.01).

### 3.7. Puerarin Exerts Anti-Inflammatory Effects in ACS Exposed Mice and CSE Stimulated HSAECs via Scavenging of ROS

**Figure 12:**
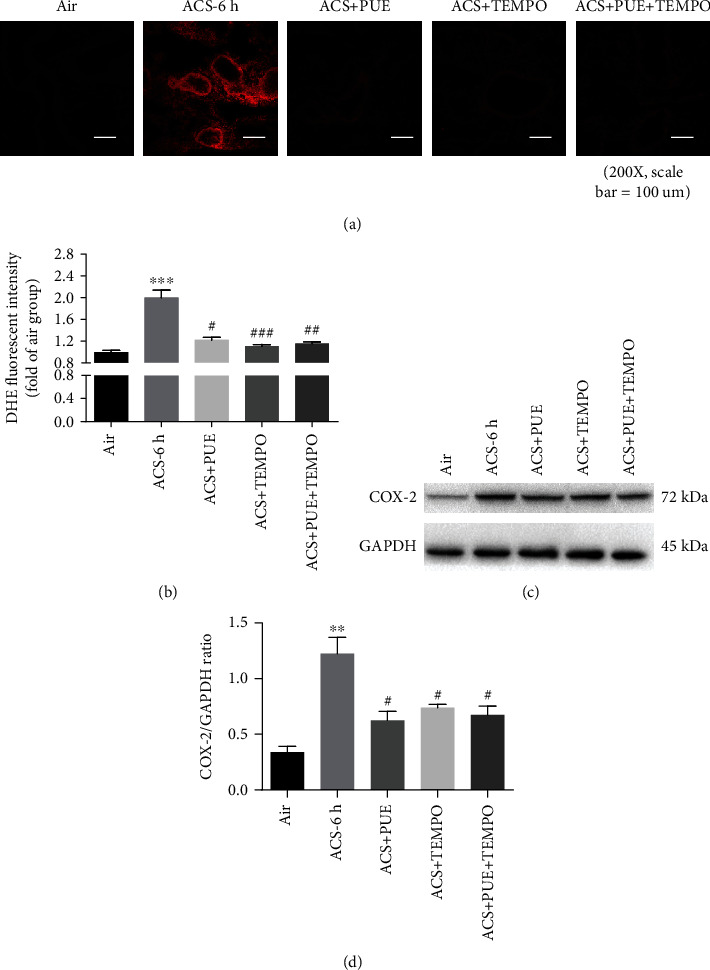
Puerarin exerts anti-inflammatory effects in ACS exposed mice via attenuation of ROS production. Mice were exposed to whole body cigarette smoking for 1 hour, and intraperitoneally injected TEMPO (100 mg/kg) and/or Puerarin (80 mg/kg) prior to and after ACS exposure, and then lung tissue harvested at 6 hours for DHE fluorescent imaging of superoxide production. Freshly isolated lung tissues were embedded in OCT, frozen at -20°C, and sectioned immediately; and lung sections incubated with DHE (5 *μ*M) for 30 min. After washing, the sections were mounted and imaged by Nikon A1+ confocal microscope. Besides, lung tissues were harvested at 6 hours after ACS exposure in mice, and lysed to examine protein expression of COX-2 by Western blotting analyses. (a, b) Representative DHE images and quantitative data of superoxide production in ACS exposed mice with or without Puerarin/TEMPO treatment. Data are show as mean ± SEM. ^∗∗∗^*p* < 0.001 vs. control/air group; ^#^*p* < 0.05, ^##^*p* < 0.01, ^###^*p* < 0.001 vs. ACS group. *n* = 3 − 8. (c, d) Representative Western blots and grouped data of COX-2 protein expression in ACS exposed mice with or without Puerarin/TEMPO treatment. Data are show as mean ± SEM. ^∗∗^*p* < 0.01vs. control/air group; ^#^*p* < 0.05 vs. ACS group. *n* = 3 − 6. PUE: Puerarin.

**Figure 13:**
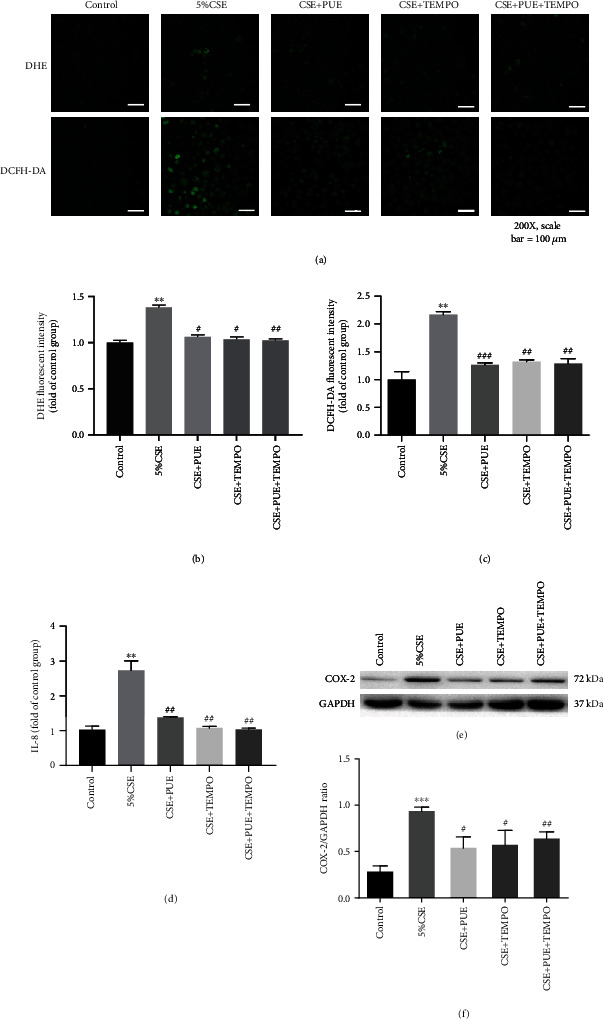
Puerarin exerts anti-inflammatory effects in CSE stimulated HSAECs via attenuation of ROS production. Human small airway epithelial cells (HSAECs) cultured in 24 well plates were exposed to 5% CSE for 6 hours in the presence or absence of TEMPO (100 *μ*M) and/or Puerarin (40 *μ*M). Cells were then incubated with DHE (2 *μ*M) or DCFH-DA (20 *μ*M) for detection of superoxide or hydrogen peroxide production, respectively. After washing, cells were mounted and imaged by Nikon A1+ confocal microscope. The images were quantified using NIH ImageJ software. (a) Representative DHE (upper panels) and DCFH-DA (lower panels) images of superoxide and hydrogen peroxide production in CSE stimulated HSAECs with or without TEMPO (100 *μ*M) and/or Puerarin (40 *μ*M) treatment. Shown are quantitative data of superoxide (b) and hydrogen peroxide (c) production in HSAECs. Data are shown as mean ± SEM. ^∗∗^*p* < 0.01 vs. control group; ^#^*p* < 0.05, ^##^*p* < 0.01, ^###^*p* < 0.001 vs. CSE group. *n* = 3 − 5. In parallel experiments, HSAECs cultured in 96-well plates were stimulated with CSE (2.5% and 5%) for 24 hours with or without TEMPO (100 *μ*M) and/or Puerarin (40 *μ*M) treatment and then cell culture medium collected for measuring IL-8 levels by ELISA in duplicate using BioTek Plate Reader. (d) Release of IL-8 in culture medium of CSE stimulated HSAECs with or without TEMPO (100 *μ*M) and/or Puerarin (40 *μ*M) treatment. Data are shown as mean ± SEM. ^∗∗^*p* < 0.01 vs. control group; ^#^*p* < 0.05, ^##^*p* < 0.01, ^###^*p* < 0.001 vs. CSE group. *n*  = 3 − 5. Additionally, HSAECs cultured in 6-well plate were stimulated with CSE (2.5% and 5%) for 6 hours with or without TEMPO (100 *μ*M) and/or Puerarin (40 *μ*M) treatment and then lysed to examine protein expression of COX-2 by Western blotting analysis. (e, f) Representative Western blots and grouped data of COX-2 protein expression in CSE stimulated HASECs with or without TEMPO (100 *μ*M) and/or Puerarin (40 *μ*M) treatment. Data are shown as mean ± SEM. ^∗∗∗^*p* < 0.001 vs. control group; ^#^*p* < 0.05, ^##^*p* < 0.01 vs. CSE group. *n* = 3 − 4. PUE: Puerarin.

Since oxidative stress provokes inflammatory responses [10], we next examined whether effects of Puerarin on attenuating inflammatory responses are mediated by inactivation of ROS production. ACS exposed mice were pretreated with superoxide scavenger TEMPO alone or in combination with Puerarin. Puerarin, TEMPO or Puerarin + TEMPO (PUE + TEM) all significantly attenuated superoxide production to control levels (Figures 12(a) and 12(b)). Further, Puerarin, TEMPO or Puerarin+TEMPO also similarly inhibited upregulation of COX-2 protein expression in ACS exposed mice (Figures 12(c) and 12(d)). Likewise, CSE exposed HSAECs were pretreated with TEMPO or in combination with Puerarin. Data indicate that Puerarin, TEMPO or Puerarin + TEMPO (PUE + TEM) all significantly attenuated superoxide and hydrogen peroxide production to control levels (Figures 13(a)–13(c)). Puerarin, TEMPO or Puerarin + TEMPO also similarly inhibited upregulation of IL-8 release and COX-2 expression in CSE stimulated HSAECs (Figures 13(d)–13(f)). Collectively, TEMPO attenuated CSE induced ROS production, IL-8 release, and COX-2 protein expression in HSAECs. The fact that Puerarin and TEMPO had similar effects, and that there is no additional synergy for combinatory treatment with Puerarin and TEMPO, indicate that the anti-inflammatory effects of Puerarin are mediated by inhibition of oxidative stress in ACS exposed mice and CSE stimulated HSAECs. Taken together, these data indicate that Puerarin inhibits inflammatory responses in ACS exposed mice and CSE stimulated HSAECs via attenuation of redox-sensitive pathways.

## 4. Discussion

The most significant findings of the present study include (1) ACS and CSE induce early oxidative stress and inflammatory responses in mice and in HSAECs respectively, which can be effectively alleviated by Puerarin administration in a dose-dependent fashion; (2) Puerarin reversal of inflammatory responses appears to be mediated by downregulation of NOX1, NOX2, and NOX4 expression in ACS exposed mice, and attenuation of NOX1 and NOX4 expression in CSE stimulated HSAECs; (3) The numbers of neutrophils and macrophages significantly elevate in both lung parenchyma and BAL fluid of ACS exposed mice, which can be completely attenuated by Puerarin administration; (4) The inflammatory mediators of TNF-*α*, KC (murine homolog of IL-8)/IL-8, NF-кB, COX-2, IL-6, and MCP-1 are markedly upregulated in ACS exposed mice and/or CSE stimulated HSAECs, all of which can be effectively abrogated by treatment with Puerarin; and (5) The anti-inflammatory effects of Puerarin are mediated by inactivation of redox-sensitive pathways in both ACS exposed mice and CSE stimulated HSAECs, since Puerarin exerted similar effects as TEMPO, and that there were no synergistic effects for combined treatment of Puerarin and TEMPO. These data establish a novel therapeutic potential of Puerarin on the oxidative and inflammatory responses at the early stage of COPD development, particularly in response to cigarette smoking exposure.

In this study, we used ACS exposed mice to investigate early oxidative and inflammatory responses during the initial stage of COPD development. Several large clinical trials targeting severe COPD have failed potentially because they were started in patients who had late stage disease that is no longer reversible [29]. Early interventions at the early “reversible stage” of the disease however may prove to be more effective. Early stage COPD is defined as COPD at GOLD stage 1 or 2. As shown in the Lung Health Study [30], smoking cessation program improved lung function after 5 years in mild and moderate COPD patients (FEV 1 > 50%). Zhou et al. [31] reported that treatment with Tiotropium, a long-acting anticholinergic bronchodilator, resulted in improved FEV1 at 24 months and ameliorated the annual decline in FEV1 in patients with early stage COPD in a multicenter, randomized, double-blind, placebo-controlled trial in China. Moreover, another important term of “early COPD” has been used to define disease stage for patients who are eversmokers (≥10 pack years smoking history), younger than 50 years, with one or more of the following: (1) FEV_1_/FVC less than lower limit of normal, (2) compatible CT abnormalities (visual emphysema, air trapping, or bronchial thickening graded mild or worse), and (3) evidence of accelerated FEV1 decline (≥60 mL/year) [32]. Some studies [32] claimed that the concept of early COPD could revolutionize understanding and therapies of the disease; although, the treatment strategies for early COPD have remained unavailable. Cigarette smoking is a major risk factor for COPD [33]. Continuous smoking and an early age at the start of smoking are significantly more prevalent in COPD patients leading to development of the disease [34]. In the present study, we used acute cigarette smoking (ACS) exposure to explore molecular mechanisms that drive cigarette smoking-induced lung inflammation at the early stage of the disease development. Besides, studies have been lacking to focus on the acute effects of smoking and the effects of Puerarin on cigarette smoking induced oxidative and inflammatory responses, and the method of cigarette smoking exposure is sometimes not unified or standardized from time to time as to how cigarette smoking is generated to induce systematic exposure in mice. Here, we used a standardized smoking machine of the TE-10B system to expose mice to ACS for 1 hour and harvested at 2, 6, and 24 hours to examine acute oxidative and inflammatory responses in mouse lungs, which mimic the pathological processes underlying development of early COPD. Then, we examined for the first time potential beneficial effects of Puerarin on the early oxidative and inflammatory responses induced by ACS, and the data indicate a robust protective role of Purerain.

In our study, superoxide production was found significantly increased in mouse lung tissues harvested 2 hours after the 1 hour ACS exposure, and most dramatically increased at 6 hours post exposure. Our data indicate a time-dependent production of superoxide in ACS exposed mouse lung tissues, which is consistent with previous observations evidencing ACS induction of superoxide and hydrogen peroxide accumulation in BAL fluid [35]. Of note, here, we elucidated that Puerarin dose-dependently abolished ACS induced superoxide production in the mouse lung tissues. Importantly, we further demonstrated that Puerain showed no effect on directly buffering away superoxide as a chemical scavenger, as determined by ESR analysis. Therefore, Puerarin is believed to exert its anti-oxidative and anti-inflammatory functions via sophisticated regulation of signaling pathways as we further showed in the study.

We next examined the potential roles of NOX isoforms in ACS induced oxidative and inflammatory activation, and data indicate that NOX1, NOX2, and NOX4 protein expression was upregulated in ACS exposed mouse lungs. These responses were markedly or completely reversed by Puerarin treatment, indicating that the protective effects of Puerarin is mediated by inhibition of the NOX isoforms to shut down ROS production. NOX family oxidases have been implicated in the pathogenesis of cardiovascular diseases [6–9]. However, the roles of NOX isoforms and NOX-derived ROS in triggering respiratory diseases have not been extensively studied, and the contributions of NOX isoforms to the development of lung emphysema remain controversial. A potential role of Puerarin in regulating expression of NOXs in lung diseases has never been studied. Importantly, combined with the ESR data, Puerarin is believed to exert protective roles via regulation of downstream signaling events, rather than inactivation of superoxide radical by chemical reaction. Therefore, our data represent first demonstration that NOX isoforms are involved during the early stage of oxidative and inflammatory responses of COPD responding to cigarette smoking exposure, which can be effectively inhibited by treatment with Puerarin. Next, we investigated the effects of Puerarin on NOXs in vitro, and data indicate that NOX1, 2, 4, and 5 were upregulated in CSE stimulated HSAECs, and the responses of NOX1 and NOX4 were attenuated by Puerarin. The differential effects of Puerarin on the expression of NOX isoforms in ACS exposed mice and CSE stimulated HSAECs (e.g., on NOX2) may be attributed to cell specific expression patterns of NOX isoforms [9], as Trocme et al. [36] reported that NOX1 is expressed in bronchial and alveolar epithelium, whereas NOX2 is mainly expressed in macrophages of emphysematous patients.

Our data systematically characterized features of inflammatory responses in ACS exposed mice, and more importantly, how these responses were regulated by Puerarin administration. Firstly, we demonstrated that neutrophils and macrophages were increased in lung tissues and BALF at 6 and 24 hours after ACS exposure in mice. Intriguingly, we observed increased lymphocytes in lung parenchyma at 24 hours after ACS exposure by H&E staining, implicating a critical role of adaptive immunity. Secondly, we observed activation of NF-кB signaling pathway and increased mRNA expression of proinflammatory cytokines (TNF-*α* and KC) in ACS exposed mice. The findings in NF-кB translocation 2 hours after ACS exposure in mice are consistent with previous notions, confirming activation of redox-sensitive transcriptional activation [37]. Of note, ACS induced ROS production mediates redox-sensitive activation of NF-*κ*B, facilitating acute and chronic inflammatory response [38]. Thirdly, we observed that the protein expression of TNF-*α* was also obviously upregulated in ACS exposed mice at 2 and 6 hours. Fourthly, our study demonstrated that protein expression of COX-2, IL-6, and MCP-1 was also significantly increased in ACS exposed mouse lungs, especially in airway epitheliums. Most importantly, we have shown that Puerarin administration exerted robust protective effects on all of these inflammatory responses observed post ACS exposure in mice.

In our study, CSE induced oxidative and inflammatory responses in HSAECs were also attenuated by Puerarin treatment. The production of superoxide and hydrogen peroxide was markedly increased in CSE stimulated HSAECs, which was substantially alleviated by treatment with Puerarin. Our data indicate markedly elevated release of IL-8 and nuclear translocation of p65 in CSE stimulated HSAECs. The COX-2 expression in CSE stimulated HSAECs was upregulated by almost 4-fold, which shares similarity with a 3.1-fold upregulation in COX-2 expression in CSE exposed HSEACs in a previous study [39]. All of these inflammatory responses in CSE stimulated HSAECs were reversed by Puerarin administration. Therefore, these data further establish beneficial effects of Puerarin on inhibiting early oxidative and inflammatory responses in CSE stimulated HSAECs, which are consistent with our findings in ACS exposed mice.

Of note, our data indicate that the early oxidative stress and inflammatory responses can be effectively reversed by Puerarin administration. This is consistent with the findings by Willemse et al. that in asymptomatic smokers who successfully quitted, inflammation in the lung (number of sputum macrophages, percentage of eosinophils and IL-8 levels) was significantly reduced; although, those already developed COPD had steady inflammatory profile after quitting smoking [40]. Indeed, acute inflammation can occur within 0-3 hrs in subjects who are exposed to acute smoking [41]. These findings again indicate importance of intervention at the early stage of COPD or early COPD, and Puerarin proves to be a robustly effective agent in attenuating early oxidative and inflammatory responses triggered by acute smoking.

Previously, TEMPO treatment or TEMPO plus Punicalagin treatment was used to examine the redox sensitivity of the protective effects of Punicalagin on hypoxia-induced hypoxic pulmonary hypertension in rats [42]. In our study, there is no additional synergy for combinatory treatment with Puerarin and TEMPO in ACS exposed mice and CSE stimulated HSAECs. Combined with the ESR data in our study indicating lack of chemical buffering of Puerarin on superoxide signal, our data indicate that Puerarin's reversal effects on oxidative and inflammatory responses are mediated by sophisticated signaling pathways involving downregulating NOX isoforms. The data confirmed a redox-sensitive attenuation of inflammatory responses by Puerarin in ACS exposed mice and CSE stimulated HSAECs, via inhibition of NOX isoforms.

Taken together, our data for the first time demonstrate robust anti-oxidative and anti-inflammatory effects of Puerarin in ACS exposed mice and CSE stimulated HSAECs. Puerarin administration downregulated protein expression of NOX1, NOX2, and NOX4 in ACS exposed mice, and that of NOX1 and NOX4 in CSE stimulated HSAECs, resulting in abrogated ROS production, alleviated inflammatory cell infiltration, and decreased expression of inflammatory mediators. These data strongly indicate that Puerarin may be used as a novel and effective therapeutic agent for early COPD or early stage COPD, via attenuation of ACS-induced early oxidative and inflammatory responses that are important mediators in the development of early or early stage COPD.

## Data Availability

All of the original data were generated originally in our research lab. We have records of all of the original data.

## Supplementary Materials

Supplementary Materials Supplemental Table 1: antibodies and dilutions used in the present study. Shown are the antibody products, reagent companies, catalogue numbers, and dilutions of antibodies used in Western blotting or immunohistochemical/immunocytochemical analyses. Supplemental Table 2: primers of KC, TNF-*α*, and *β*-actin used for RT-PCR analyses in the present study. RT-PCR was used to determine mRNA expression of inflammatory mediators. Total RNA of mouse lung tissues was isolated using TRIzol Reagent and reversed to cDNA using High-Capacity cDNA Reverse Transcription Kit. Subsequently, PCR was performed using SsoFast Evagreen supermix on a CFX96 Real-Time System according to the manufacturer's instructions. Supplemental Table 3: donor information for human small airway epithelial cells (HSAECs) purchased from Lonza Biosicence. Three independent batches of HSAECs were purchased from Lonza Bioscience, which were isolated from three healthy donors. Shown are donor information including lot number, manufacture date, age, sex, and race. HSAECs were cultured in the small airway growth medium (SAGM) supplemented with growth factors following the manufacturer's instructions.
