# Supplementary material for: Reversal of NADPH Oxidase-Dependent Early Oxidative and Inflammatory Responses in Chronic Obstructive Pulmonary Disease by Puerarin
Source: Oxid Med Cell Longev. 2022 Apr 25;2022:5595781. doi: 10.1155/2022/5595781 (PMC9151001; doi:10.1155/2022/5595781)
Supplement: Supplementary Materials — Supplemental Table 1: antibodies and dilutions used in the present study. Shown are the antibody products, reagent companies, catalogue numbers, and dilutions of antibodies used in Western blotting or immunohistochemical/immunocytochemical analyses. Supplemental Table 2: primers of KC, TNF-α, and β-actin used for RT-PCR analyses in the present study. RT-PCR was used to determine mRNA expression of inflammatory mediators. Total RNA of mouse lung tissues was isolated using TRIzol Reagent and reversed to cDNA using High-Capacity cDNA Reverse Transcription Kit. Subsequently, PCR was performed using SsoFast Evagreen supermix on a CFX96 Real-Time System according to the manufacturer's instructions. Supplemental Table 3: donor information for human small airway epithelial cells (HSAECs) purchased from Lonza Biosicence. Three independent batches of HSAECs were purchased from Lonza Bioscience, which were isolated from three healthy donors. Shown are donor information including lot number, manufacture date, age, sex, and race. HSAECs were cultured in the small airway growth medium (SAGM) supplemented with growth factors following the manufacturer's instructions. [file 5595781.f1.docx]

Table1. **Antibodies and dilutions used in the present study**. Shown are the antibody products, reagent companies, catalogue numbers, and dilutions of antibodies used in Western blotting or immunohistochemical/immunocytochemical analyses.

| Antibody | Company | Cat. | Dilution (WB, unless noted) |
| --- | --- | --- | --- |
| NOX1 | NOVUS biological | NBP1-31546 | 1:1000 |
| NOX2 (gp91phox) | SANTA CRUZ BIOTECHNOLOGY | sc-130543 | 1:100 |
| NOX4 | NOVUS biological | NB110-58849 | 1:500 |
| NOX5 | ABCAM | ab191010 | 1:500 |
| Phospho-NF-kB p65 (Ser536) | CST | #3033 | 1:500 (IHC) |
| COX-2 | Cayman chemical canpany, | NO.160106 | 1:200  1:50 (IHC) |
| IL-6 | ABCAM | ab208113 | 1:400 (IHC) |
| MCP1/CCL2 | NOVUS biological | NBP1-07035 | 1:500 (IHC) |
| TNF alpha | Abcam | ab6671 | 1:500 |
| GAPDH | Abcam | ab181602 | 1:10000 |
| β-actin | Abcam | Ab20272 | 1:5000 |
| NF-κB p65 | CST | #8242 | 1:500 (IHC and ICC) |
| Anti-rabbit IgG secondary antibody | Thermo fisher scientific | 65-6120 | 1:5000 |
| Anti-mouse IgG secondary antibody | Thermo fisher scientific | 62-6520 | 1:5000 |
| Goat Anti-Rabbit IgG H&L (Alexa Fluor® 555) | ABCAM | ab150078 | 1:500 |

Table 2. **Primers of KC, TNF-α and β-actin used for RT-PCR analyses in present study.** RT-PCR was used to determine mRNA expression of inflammatory mediators. Total RNA of mouse lung tissues was isolated using Trizol reagent, and reversed to cDNA using High-Capacity cDNA Reverse Transcription Kit. Subsequently, PCR was performed using SsoFast Evagreen supermix on a CFX96 Real-Time System according to the manufacturer’s instructions.

| KC forward | ACCCAAACCGAAGTCATAGCC |
| --- | --- |
| KC reverse | TTGTCAGAAGCCAGCGTTCA |
| TNF-α forward | TCTGTCTACTGAACTTCGGGGTGA |
| TNF-α reverse | TTGTCTTTGAGATCCATGCCGTT |
| β-actin forward | CAC TGT GCC CAT CTA CGA |
| β-actin reverse | GTA GTC TGT CAG GTC CCG |

Table 3. **Donor information for human small airway epithelial cells (HSAECs) purchased from Lonza Biosicence.** Three independent batches of human small airway epithelial cells (HSAECs) were purchased from Lonza Bioscience, which were isolated from three healthy donors. Shown are donor information including lot number, manufacture date, age, sex and race. HSAECs were cultured in the small airway growth medium (SAGM) supplemented with growth factors following the manufacturer’s instructions.

| \| Lot Number \| \| --- \| | 18TL179344 | 18TL082942 | 18TL127525 |
| --- | --- | --- | --- | --- |
| Manufacture Date | 10-Jul-18 | 9-Apr-18 | 14-May-18 |
| Age | 25Y | 68Y | 30 Y |
| Sex | FEMALE | FEMALE | FEMALE |
| Race | Caucasian | Hispanic | Hispanic |
